# Development of a self-administered web-based test for longitudinal cognitive assessment

**DOI:** 10.1038/srep19114

**Published:** 2016-01-08

**Authors:** Luis Ruano, Andreia Sousa, Milton Severo, Ivânia Alves, Márcio Colunas, Rui Barreto, Cátia Mateus, Sandra Moreira, Eduardo Conde, Virgílio Bento, Nuno Lunet, Joana Pais, Vítor Tedim Cruz

**Affiliations:** 1 Department of Neurology, São Sebastião Hospital, Entre Douro e Vouga Hospital Center, 4520-211 Santa Maria da Feira, Portugal; 2 Department of Clinical Epidemiology, Predictive Medicine and Public Health, University of Porto Medical School, 4200-319 Porto, Portugal; 3 EPIUnit - Institute of Public Health, University of Porto, 4050-600, Porto, Portugal; 4 Clinical Research Office, Health Sciences Department, University of Aveiro, 3810-193, Aveiro, Portugal; 5 University Institute of Maia – ISMAI, 4475-690, Maia, Portugal

## Abstract

Sequential testing with brief cognitive tools has been recommended to improve cognitive screening and monitoring, however the few available tools still depend on an external evaluator and periodic visits. We developed a self-administered computerized test intended for longitudinal cognitive testing (Brain on Track). The test can be performed from a home computer and is composed of several subtests, expected to evaluate different cognitive domains, all including random elements to minimize learning effects. An initial (A) and a refined version of the test (B) were applied to patients with mild cognitive impairment or early dementia (n = 88) and age and education-matched controls. A subsample of a population-based cohort (n = 113) performed the test at home every three months to evaluate test-retest reliability. The test’s final version Cronbach’s alpha was 0.90, test scores were significantly different between patients and controls (*p* = 0.001), the area under the receiver operating characteristic curve was 0.75 and the smallest real difference (43.04) was lower than the clinical relevant difference (56.82). In the test-retest reliability analysis 9/10 subtests showed two-way mixed single intraclass consistency correlation coefficient >0.70. These results imply good internal consistency, discriminative ability and reliability when performed at home, encouraging further longitudinal clinical and population-based studies.

The timely identification of cognitive deficits can be crucial to guide therapeutic intervention^1^, cognitive training^2^,^3^ and functional rehabilitation^4^ in patients with neurodegenerative disorders, cerebrovascular dementia and young patients with central nervous system (CNS) diseases such as multiple sclerosis (MS) and traumatic brain injury. The standard for cognitive assessment relies on an extensive evaluation of multiple cognitive domains by a trained professional^5^. These neuropsychological test batteries have a high sensitivity and specificity for the detection of dementia^6^; however, their application is time and resource consuming and therefore not a practical strategy for cognitive screening in the general population or for monitoring cognitive function in patients with CNS diseases. Brief low-cost tools have been developed for these aims, but mostly lack the desired discriminative ability to predict the progression to dementia^7^. As the clinical definition of dementia implies a significant decline in performance from a pre-morbid cognitive function, some authors have suggested that cognitive measurements should record alterations in state, rather than the current state^8^. Accordingly, follow-up cognitive testing has been recommended to improve the diagnostic reliability for mild cognitive impairment (MCI)^5^ and to monitor cognitive deterioration in patients with MS^9^. However, few screening tools have been clinically validated for this purpose, and still depend on a trained external evaluator and periodic clinical visits^10^,^11^. A self-administered web-based cognitive test that could be repeated periodically would present some advantages to address these issues. Namely, it could be performed at home, therefore being more cost-effective and convenient for the patient, allow the use of random elements and alternate sequences to minimize learning effects, and offer the possibility of adapting the testing difficulty to the baseline cognitive performance of the patient. A strategy based on such a tool could be useful for the screening of patients with subjective memory complaints in primary care and to monitor patients with CNS diseases at risk for cognitive deterioration. It could also prove useful to identify patients in prodromal phases of progressive neurodegenerative diseases to enroll in clinical trials.

Computerized cognitive tests have existed for several decades, they have several known advantages for use in clinical and research settings: the reduced costs, the ability to accurately measure and store test responses and latency times, the minimization of examiner subjectivity and the potential for multiple test versions, allowing for adaptive testing^12^,^13^. Some of the existing computerized batteries have already shown an overall reliability and discriminative ability comparable to traditional neuropsychological testing^12^,^13^. There are also potential limitations for computerized cognitive testing, namely the effect of previous experience with computer interfaces on test response^14^ and a perceived lack of adequately established psychometric standards^15^.

Most of the existing computerized cognitive tests have been designed to mirror the comprehensive neuropsychological assessment batteries^15^, applied by a trained professional in a clinical setting and not designed for screening or monitoring cognitive impairment^16^. In recent years, several shorter cognitive tools aimed at screening for cognitive impairment have been developed; nevertheless most of them still require a health professional to be started. To the best our of knowledge, none has been specifically designed for longitudinal use^13^,^17^. Therefore, we aimed to develop a web-based self-administered test intended for longitudinal cognitive screening and monitoring.

## Methods

### Rationale and principles for test development

The Brain on Track test was designed to take full advantage of the features and flexibility of a web-based interface, rather than to replicate the existing pen and paper cognitive tests. As an initial base for subtest development, we used simple computerized cognitive training exercises from an existing online platform (Cogweb), being developed by elements from the same research team since 2005^18^. This web-based platform includes more than 60 cognitive training exercises that target different cognitive domains, allowing for remote cognitive training programs in the patient’s living environment. These exercises already passed through extensive usability testing in a wide spectrum of ages and disease models, and it was demonstrated that patients could use them independently and repeatedly from their home computers^18^. We expected that exercises based on goal-oriented tasks would have some advantages as a model for computerized self-administered cognitive subtests. This task-based structure could allow for a better understanding of the objective of each subtest and it would motivate the patient to perform at his/her best level. The stimuli in the subtests were optimized through an iterative process; most of them include simultaneous visual and audio cues, all were designed with high contrast graphics and large font sizes. A pool of 50 potential subtests, most of them adapted from the existing Cogweb exercises, was initially developed. As the Brain on Track was intended to be used repeatedly, random elements and sequences were used to minimize learning effects. All of the subtests include at least a random element in each task or compose of multiple predefined similar tasks that are randomly selected and ordered for each trial. For example, in the Opposite subtest, the participant must press the keyboard arrow in the opposite direction to that shown by a large arrow on screen; the direction of the arrow is randomly generated. Another example, in the Puzzles subtest, there are 40 alternate puzzles of similar design and difficulty; the puzzle selection and order is randomly generated at the start of each trial. Furthermore, the subtests were designed with several versions with different levels of difficulty, to offer the possibility of adaptive testing. Each subtest begins with a set of written instructions that are shown on the screen and read by a pre-recorded voice and has a limited duration of two minutes, including the tests instructions. During that time, the participant must perform the tasks described in Appendix 1. The number of tasks presented to the participant within each subtest is limited only by the time limit. The subtest score is the number of tasks performed correctly in each subtest and varies from 0 to the maximum number of tasks the participant can perform successfully within the time limit.

The subtests were designed and programmed to be light on data usage transmitted over the web and of local computer resources. Before the start of each subtest, the data needed for its completion is loaded into the local browser; only then is the participant able to signal using a dialog box if he/she is ready to start. The system was tested and optimized to work in the different versions of the four most used browsers (Google Chrome, Internet Explorer, Firefox and Safari).

The studies reported in this paper have been approved by the appropriate ethics committees. The web-based system for data collection has been approved by the Portuguese Data Protection Authority. All of the data transmission was encrypted, there was no personal data transmission over the Internet and the participants’ identity information was stored in a separate off-line database. All of the participants in the studies gave their informed consent prior to their inclusion; in participants with cognitive impairment the caregiver consent was also requested. The statistical package used was SPSS Statistics 22.0.

### Development of the Brain on Track test (Study I)

#### Development of the first test version – Test A

From the initial pool of 50 potential subtests, a group of experienced neuropsychologists and neurologists defined and developed a group of 9 subtests, expected to evaluate attention, memory, executive functions, language, constructive ability and spatial processing (Test A), with a total duration of 18 minutes (Word categories task, Attention task II, Sequences, Visual memory task I, Puzzles, Written comprehension, Shopping task, Verbal memory task, Inhibitory control). In the subtest development, we identified the use of the keyboard for word input as a major difficulty in the elderly, being more dependent on the level of previous computer experience than the use of the mouse, and a common source of error through input mistakes when the participant performed the test autonomously. For these reasons, the subtest interface was designed to depend solely on the mouse or on pressing unique keyboard keys, therefore the episodic verbal memory subtest is based on cued recall and a verbal fluency subtest was not included. Subtest description can be found in Appendix 1.

For a cognitive test in the adult population, the minimal important clinical difference (MID) that should be detected corresponds to the change from healthy status to early stage cognitive disorders with clinical complaints. Therefore, we applied Test A in two groups: 1) consecutive patients referred to a memory clinic with mild cognitive impairment (MCI) or early stage (mild) dementia; 2) a convenience sample of community controls, matched for age group (±10 years) and educational attainment level (groups: 1–4; 5–9; 10–12; and >12 years) recruited from adult learning centers in the hospital region, healthy hospital volunteers, patient relatives and health workers.

The overall inclusion criteria for patients were: ≥18 years of age and no physical impairment precluding using a computer and mouse interface (Table 1). Mild cognitive impairment was defined as the presence of subjective cognitive complaints over a period of at least 6 months reported by the patient or family members, one cognitive domain 1.5 standard deviations (SD) or more below age-corrected norms in the neuropsychological evaluation, without clinical depression and without impairment in daily activities^19^. For mild dementia, the inclusion criteria were fulfilling the DSM-V definition for major neurocognitive disorder^20^ (significant cognitive impairment in at least one cognitive domain representing a significant decline from a previous level of functioning that interferes with independence in everyday activities) and a mild dementia, defined as a score of 1.0 in the clinical dementia rating scale^21^ (Table 1). The initial clinical classification was confirmed after at least 6 months of clinical follow-up by a neurologist. Every patient had a complete diagnostic workup, including blood analysis for treatable causes of dementia, imaging studies and a complete neuropsychological evaluation.

The inclusion of community controls was determined based on an interview with a neurologist and a review of previous medical history. The inclusion criteria were: ≥18 years of age, absence of any neurological, psychiatric or systemic disease that could impair cognition (except for stable depressive symptoms), absence of drug use that could impair cognition in the past 3 months, absence of alcohol or substance abuse in the previous 2 years, no physical impairment precluding the use of a computer and mouse interface and no subjective memory complaints.

The tests were self-administered in a hospital clinic, under the observation of a member from the research team. Difficulties in understanding or performing the tests and failure to complete the test battery were systematically registered.

#### Refinement to the second test version – Test B

A second version of the test (Test B) was defined after analysis of the results from Test A, retaining 7 subtests from Test A and introducing 5 new subtests (Word categories task, Attention task I, Auditory memory task, Opposite task, Visual memory task II, Attention task II, Sequences, Calculus task, Visual memory task I, Puzzles, Written Comprehension, Shopping Task; subtest description can be found in Appendix 1). Test B was self-administered by a group of patients and matched controls, using the same study protocol, setting and inclusion criteria already described for test A.

#### Statistical analysis

Principal component analysis was performed to evaluate dimensionality of the subtests^22^. The acceleration factor that corresponds to the numerical solution to the elbow of the scree plot was used to define the number of components retained. Subtests with high factor loading (factor loading >0.50) were retained. The internal consistency was assessed using Cronbach’s alpha to discard subtests that lowered the overall internal consistency and/or with lower item-total correlation (<0.50)^23^.

The subtest scores were standardized to a t-score using the mean and standard deviation of the healthy controls as the reference. The final test scores are the total sum of the subtests’ scores. To compare the differences in age, education and test scores between the two groups Student’s T test for independent samples was used, since all variables presented a normal distribution (p > 0.05 in Kolmogorov–Smirnov test). Linear regression analysis was used to assess the correlation of the Brain on Track test scores to the scores of the Montreal Cognitive Assessment (MoCA) and the Mini Mental State Examination (MMSE). A multivariable linear regression model was used to identify a possible effect of age, gender and education on test scores, independent of test groups (patient vs. control, and their potential interactions with the test group). To estimate the predictive accuracy of test scores to distinguish between patients and controls and calculate the areas under the corresponding receiver operating characteristic curves (AUC)^24^, logistic regression models were fitted using test group (patient vs. control) as the dependent variable, test scores as the independent variable and adjusting for factors associated to test scores (age, education, and interaction between education and test scores).

Considering the use of Brain on Track to detect cognitive impairment in the adult population, the minimal relevant status change to be detected can be defined as the difference between healthy individuals and patients when first presenting with memory complaints caused by early stage cognitive disorders. By selecting the two test groups that fit these criteria (healthy controls vs. patients with early stage cognitive impairment), we estimated the MID as the difference in the average test score between these two groups. To assess the test ability to detect clinically important changes over time, the difference between the MID and the smallest real difference (SRD) was calculated^25^,^26^. The SRD was estimated using the following formula: SRD = Standard Error of Measurement (SEM)*1.96. The SEM was calculated as SEM = SD*√(1–Cronbach’s alpha)^27^.

To validate the test, we hypothesized that the older, less educated and the patients affected with MCI/early dementia would have lower scores. For the comparison between patients and controls, we hypothesized that the test would have at least an acceptable predictive ability to detect MCI/Mild dementia in a single use (AUC≥0.70) and, more importantly for a repeatable test, that it would be sensitive to status change (SRD < MID).

### Test-retest from home (Study II)

#### Design

The refined version of Test B with the 8 subtests retained after Study I (Word categories task; Opposite task; Visual memory task II; Attention task II; Sequences; Calculus task; Puzzles and Written comprehension) was used to assess test-retest reliability, with the test being self-applied at home in a sub-sample of participants from the EpiPorto cohort. The EpiPorto cohort was assembled between 1999 and 2003, as a representative sample of adult (≥18 years) community dwellers of Porto, an urban center in the northwest of Portugal, with approximately 300,000 inhabitants at that time^28^. Households were selected by random digit dialing of landline telephones. Within each household, a permanent resident aged 18 years or more was selected by simple random sampling. The initial number of participants in the cohort was 2485 (70% participation). In the 2013–2014 revaluation of the EpiPorto cohort, the first 300 consecutive participants were invited to participate in the Brain on Track test-retest study. The inclusion criteria were MoCA scores above the cut-point stratified by age and educational attainment for the Portuguese population (1.5 SD below the mean)^29^ to exclude participants with cognitive impairment, access to a computer at home, and being able to use a computer and mouse interface without external help (Table 1).

After accepting to participate, each participant performed the test under the supervision of an element from the research team in a clinical lab. This session had two main goals: a) teaching the participant how to login to the Brain on Track web page and accustoming the participant with the user interface and b) guaranteeing that the participant understood the instructions and mechanics of each game, so that subsequent testing would not be as dependent on learning effects.

One week after the training session, the participants were asked to perform the test at home by e-mail and SMS. The participants accessed the web site from their home computer and performed the testing autonomously. They were asked to repeat the test a 2^nd^ time, 3 months later, and a 3^rd^ time 6 months after the first trial.

#### Statistical analysis

Test-retest reliability for each subtest was assessed using consistency two way mixed single intraclass correlation coefficient (ICC)^26^,^30^. We hypothesized that most of the subtests would have good test-retest reliability (minimum ICC of 0.70). Additionally, learning effects between trials were also tested using Student’s T test for related samples, since all variables presented a normal distribution (p > 0.05 in Kolmogorov–Smirnov test). Ethical approval of research: Study I was approved by the ethics committee of Hospital São Sebastião, Centro Hospital de Entre o Douro e Vouga, Santa Maria da Feira, and Study II by the ethics committee of Hospital de São João, Porto and the methods were conducted in accordance with the approved guidelines. All patients and caregivers were provided with information about the purpose and procedures of the study and provided written informed consent.

## Results

### Study I

A total of 176 individuals were recruited for Study I, 98 performed Test A (49 patients and 49 controls), 78 performed Test B (39 patients and 39 controls). There were no significant differences between patients and controls regarding age and education (Table 2). Participants that performed Test B were older, with a significant difference (mean age difference = 4.52 years; *t*(174) = −2.97; *p* = 0.003), and slightly less educated, but with a non-significant difference (average education difference = 0.51 years; *t*(174) = −1.29; *p* = 0.200).

In the principal component analysis the solution defined by the scree plot criteria was of one principal component in both tests. For Test A the eigenvalue for the first factor was 5.49, corresponding to 55.8% of the explained variance, the second factor had an eigenvalue value of 1.39, corresponding to 11.6% of the explained variance. Therefore, one principal component was defined, including the subtests Word categories task, Attention task II, Sequences, Puzzles, Written comprehension, Shopping task, Verbal memory task, Inhibitory control; one subtest (Visual memory task I) did not reach the predefined factor loading of 0.50 and was discarded (Table 3). For Test B the eigenvalue for the first factor was 5.87, corresponding to 55.8% of the explained variance, the second component had an eigenvalue value of 8.8, corresponding to 9.8% of the explained variance. Therefore, one principal component was defined, including the subtests Word categories task, Attention task I, Auditory memory task, Opposite task, Visual memory task II, Attention task II, Sequences, Calculus task, Visual memory task I, Puzzles, Written Comprehension, Shopping Task; two subtests (Attention task I and Auditory memory task; Table 4) did not reach the predefined factor loading of 0.50 and was discarded (Table 3).

Concerning internal consistency, the subtests retained after principal component analysis from Test A had good internal consistency (Table 3), but one subtest from Test B (Visual memory task I) did not meet the predetermined standard (Table 4). The final versions of the two tests showed high internal consistency, with Cronbach’s alpha of 0.91 for Test A and 0.90 for Test B.

The average score for Test A was 9.03 (95% Confidence Interval (CI): −7.20; 25.26) in patients with MCI/Mild Dementia and 50.00 (95%CI: 31.49; 68.50) in controls, showing a significant difference (*t*(96) = 3.35; *p* = 0.001; Table 5). For Test B, the average scores were −6.56 (95%CI: −34.96; 21.84) in MCI/Mild Dementia and 50.00 (95%CI: 27.50; 75.52) in controls, also with a significant difference (*t*(76) = 3.16; *p* = 0.02). There was a moderate to strong positive statistically significant correlation between Brain on Track test scores and the test scores from MoCA (Test A: *p* < 0.001; R = 0.52 *β* = 0.04 (95% CI: 0.03; 0.06); Test B: *p* < 0.001; R = 0.62 *β* = 0.03 (95% CI: 0.02; 0.04): and MMSE (Test A: *p* < 0.001; R = 0.39 *β* = 0.02 (95% CI: 0.01; 0.02); Test B: *p* < 0.001; R = 0.52 *β* = 0.02 (95% CI: 0.01; 0.03).

**Figure 1:**
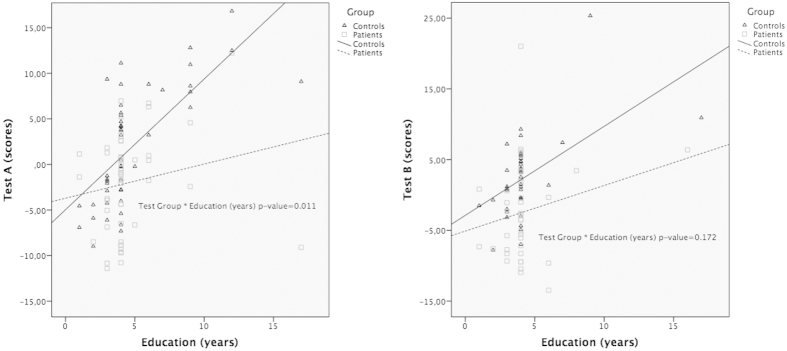
Association between test scores and education by test group (Controls vs. MCI/Mild Dementia) in Test A and Test B.

In the linear regression analysis, there was a significant association between the test scores and age, Test A: *p* < 0.001; *β* = −0.20 (95% CI: −0.29; −0.11); Test B: *p* = 0.041; *β* = −0.20 (95%CI: −0.38; −0.01) and also between the test scores and educational attainment, Test A: *p* = 0.001; *β* = 0.67 (95% CI: 0.30; 1.04); Test B: *p* = 0.007; *β* = 0.80 (95% CI: 0.29; 1.37), while no significant effect was identified for gender (Test A: β = −2.86 *p* = 0.78; Test B: β = 9.20 *p* = 0.591). In Test A, a significant interaction was found between test group (patient vs. control) and educational attainment: in the more educated individuals the differences in test scores between test groups are higher than in those with lower education levels (*p* = 0.011; *β* = −0.89 (95%CI: −1.57; −0.21)) (Fig. 1). Although a similar trend can also be observed in Test B (Fig. 1), the interaction was not statistically significant (*p* = 0.172; *β* = −0.76 (95%CI: −1.87; 0.34)).

To estimate the predictive accuracy of the test to distinguish between patients and controls, and using a logistic regression model adjusted for the parameters associated with test scores (age, education and the interaction between age and education), the AUC was 0.741 for Test A and 0.753 for Test B.

The smallest real difference (SRD) between test groups was 37.89 for Test A and 43.04 for Test B (Table 5), lower than the predefined clinically relevant differences (MID) for both tests (4.00 and 4.82 respectively). The difference between the SRD and MID was higher in Test B (22.9%) than in Test A (7.5%).

The Verbal Memory and Inhibitory Control subtests presented a floor effect in the control participants with lower to average education and were perceived to be the most difficult subtests from Test A by the participants and neuropsychologists. For this reason, and given the worst discriminative ability of Test A in patients, they were replaced by simpler alternatives in Test B: the Verbal memory task was replaced by the Auditory memory task and the Visual memory task II as alternative tests for episodic memory; the Inhibitory Control task was replaced by the Opposite task and the Calculus tasks as alternative tests for inhibitory control/executive function. Difficulties in understating the goal were reported by some patients and controls in two of the subtests from Test B (Shopping task and Visual Memory task I), so these tests were excluded from the refined version of Test B in which the test-retest analysis was completed. The remaining test instructions and mechanics were well understood by the patients and controls.

### Study II

From the 300 potential participants, 63 (21%) were not eligible because of MoCA test scores below the defined cut-point. From the remaining participants, 73 (24%) were excluded because they did not have continuous access to a computer at home, 18 (6%) could not use a computer and mouse interface without external help and 17 (6%) refused to participate in the test-retest study from home. The study was initiated by 129 participants, from whom 113 completed the 3 trials at home (87.6%). The mean (SD) age and years of schooling of the study participants were 64.8 (6.0) and 11.8 (4.6). There was a slight upward trend in subtests scores (Table 6), which was statistically significant between trials 2 and 3 of the Opposite (*t*(112) = 2.89; *p* = 0.005; mean difference = 1.08; sd = 2.30) and the Written Comprehension (*t*(112) = 3.03; *p* = 0.003; average difference = 0.87; sd = 2.3) tasks. In the analysis of the test-retest reliability for 3 consecutive trials, only 1 subtest showed a low intraclass correlation (Attention task II), all of the other subtests showed high ICC, with 6 of 10 tests with ICC higher than 0.80 (Table 6).

## Discussion

In this paper, we describe the assembling of Brain on Track, a web-based self-administered test intended for longitudinal cognitive testing, and present the results of its early validation process. The second version (Test B) was able to improve the initial version (Test A) and showed good internal consistency, reproducibility, positive correlation with existing cognitive screening tests and ability to identify clinically relevant differences. The subtests showed high test-retest reliability when performed at home, notwithstanding a small learning effect between trials was identified in some subtests. Future longitudinal studies with longer follow-up will allow us to address the potential impact of additional trials in learning effects and test-retest reliability. The education level of patients in this study is lower than what is usually found in the literature. This is not surprising, given that the Portuguese population is one of the least educated in Europe, especially in the elderly groups^31^. The fact that the Brain on Track test could be successfully applied in this setting underlines its potential as an inclusive tool for cognitive testing. On the other hand, this could also represent a potential limitation for the generalization of the test to more educated populations. However, the differences in test scores between patients and controls increased among the more educated when compared with the least educated. Consequentially, the predictive accuracy is higher in the more educated group.

The Brain on Track tool shares potential advantages with the other computerized cognitive tests: the cost-effectiveness, the ability to accurately record and store the responses, the minimization of examiner bias and the potential for adaptive testing^12^,^13^. The main criticisms of these tools relate to the lack of adequately established psychometric standards and the potential difficulties in the response for older adults unfamiliar with computer interfaces^12^,^13^. In a population-based cohort of adult individuals in Portugal, 70% of could be included in this strategy and 87.6% of these participants were able to complete 3 test sessions without external help, suggesting a good usability in this setting. We expect the resistance and lack of familiarity with computers to decrease in the near future, as the number of adults with access and experience in computer use increases. Performing the tests at home without supervision also creates the potential issue of non-compliance, which if not controlled could represent a potential limitation. The usability and the impact of non-compliance will be explored in future studies with larger groups of patients and healthy controls using qualitative interviews and focus groups with patients and relatives and by alternating observed and non-observed testing sessions in the long-term monitoring plan.

There are some major technological hurdles in the development process of computerized tests performed at home that can become potential limitations if not properly addressed, namely the different hardware, software and Internet speed of the patients’ computers. We are confident we were able to minimize their impact on test results by 1) using web-based instead of locally installed software, allowing to control the subtest duration and the latency times in real time and thus guarantying homogeneity in the different platforms and 2) preloading all of the data needed for each subtest before its initiation, resulting in Internet speed affecting the waiting time between the subtests, but not the duration of each subtest, nor the latency between the tasks within each subtest.

Notwithstanding all of the challenges their implementation entails in the real world, computerized cognitive tests can present a diagnostic accuracy comparable to traditional neuropsychological testing^12^,^13^. In the last decade, the field has increasingly expanded in the direction of shorter screening tests^13^, able to address the unmet need for a cost-effective diagnostic approach for the increasing number of individuals at risk for dementia in the general population. Several such tests have shown good diagnostic accuracy and have entered clinical use, such as the National Institutes of Health Toolbox Cognition Battery^32^, the CogState^33^ and the Cognitive Stability Index^34^. However, while these tests can replace the existing pen and paper screening tests like MoCA and MMSE with some potential advantages, they still require a trained evaluator and a patient visit to a clinic. Other approaches for expanding the accessibility to cognitive screening have been proposed, namely the Audio Recorded Cognitive Screen^35^, that relies on an audio recording to provide testing instructions and can be applied without an external evaluator, though its use was not yet validated for repeatable testing or for remote self-administration. In the last years, a few cognitive tests have been developed and validated that allow for self-administration and remote testing, such as the Computer Assessment of Mild Cognitive Impairment^36^, MicroCog^37^ and COGselftest^38^.These tests, like the Brain on Track test, showed good neuropsychological parameters when the tests were conducted in a clinical setting, but they were primarily designed for single use and are not validated for longitudinal follow-up^13^,^17^. For a first use of the Brain on Track test in a longitudinal screening strategy (i.e.: patients with memory complaints in primary care), a cut-point optimized for positive likelihood ratio could be defined (specificity = 0.90; sensitivity = 0.54; positive likelihood ratio = 4.73; negative likelihood = 0.46) and patients falling below would be classified as probably affected and referred to a neurologist. It is important to emphasize that the AUC and other discriminative statistics can serve as proof of concept for the test’s discriminative ability but they do not accurately assess the test performance for repeated use; the positive difference between the SRD and MID and the test-retest reliability of the Brain on Track test are good indicators that the tool will be able to identify this cognitive decline over time. To the best of our knowledge, this is the first development process for a computerized repeatable cognitive test where test selection was performed based on the test-retest reliability from home. Moving forward to longitudinal validation, we plan to test different strategies to identify possible cognitive impairment: 1) test scores falling bellow an expected performance threshold for each age/education group and 2) a pattern of decline in individual performance.

These initial results are encouraging and validate the Brain on Track test as a valid cognitive test. The undergoing clinical based and population longitudinal studies will allow for further development, refinement and validation for longitudinal clinical use.

## Additional Information

**How to cite this article**: Ruano, L. *et al.* Development of a self-administered web-based test for longitudinal cognitive assessment. *Sci. Rep.* **6**, 19114; doi: 10.1038/srep19114 (2016).

## Supplementary Material

Supplementary Information

## Figures and Tables

**Table 1:** Inclusion criteria for participants.

**All participants**• ≥18 years of age• No physical impairment precluding using a computer and mouse interface	
**Study I**	
**Patients**	**Controls**
Mild cognitive impairment• Subjective cognitive complaints over a period of at least 6 months• One cognitive domain 1.5 standard deviations (SD) below norm in the neuropsychological evaluation• No clinical depression• No impairment in daily activities**Or**Mild dementia• Complying DSM-V criteria for major neurocognitive disorder^20^ (significant cognitive impairment in at least one cognitive domain representing a significant decline from a previous level of functioning that interferes with independence in everyday activities)• Score of 1.0 in the clinical dementia rating scale^21^	• Absence of any neurological, psychiatric or systemic disease that could impair cognition (except for stable depressive symptoms)• Absence of drugs that could impair cognition in the past 3 months• Absence of alcohol or substance abuse in the previous 2 years• No subjective memory complaints
**Study II**• Montreal Cognitive Assessment scores above the cut point stratified by age and educational attainment for the Portuguese population (1.5 SD below mean)^29^• Access to a computer at home• Being able to use a computer and mouse interface without external help	

**Table 2:** Participant demographics and cognitive screening test scores.

	Age	Education	MMSE	MoCA
	Mean (Standard Deviation), *years*		Mean (Standard Deviation), *test scores*	
Test A				
Controls (n = 49)	67.9 (11.9)	4.9 (3.0)	27.8 (1.7)	21.9 (3.2)
MCI/Mild Dementia (n = 26/n = 23)	68.2 (11.8)	4.6 (2.6)	26.4 (3.3)	17.3 (5.6)
p-value (Student’s T test)	0.90	0.60	0.02	0.01
Test B				
Controls (n = 39)	72.2 (7.2)	4.1 (2.5)	25.8 (2.5)	19.4 (3.7)
MCI/Mild Dementia (n = 18/n = 21)	73.0 (7.5)	4.2 (2.4)	24.0 (3.8)	15.0 (4.5)
p-value (Student’s T test)	0.64	0.89	0.03	0.001

MCI – mild cognitive impairment; MoCA – Montreal Cognitive Assessment; MMSE – Cognitive Assessment and Mini Mental State Examination.

**Table 3:** Principal components analysis and reliability analysis for Test A.

	Correct answers Mean (Standard Deviation) *% Correct response*			Principal component analysis	
	Patients	Controls	Reliability analysis	Corrected Item-Total Correlation	Cronbach’s Alpha if Item Deleted
Word categories task	8.24 (4.49) *81.4%*	11.59 (4.03) *92.1%*	0.818	0.744	0.888
Attention task II	19.94 (10.79) *93.7%*	22.16 (7.79) *98.0%*	0.698	0.614	0.900
Sequences	5.24 (3.53) *88.0%*	8.80(4.16) *97.0%*	0.777	0.704	0.892
Visual memory task I	5.59 (2.68) *84.8%*	6.22 (1.82) *93.6%*	0.495^A^	–	–
Puzzles	2.00 (1.37) *100%*	2.71 (1.79) *100%*	0.693	0.614	0.900
Written comprehension	14.18 (3.22) *88.0%*	15.35 (3.49) *97.9%*	0.789	0.717	0.891
Shopping task	3.90 (2.88) *88.0%*	6.29 (3.22) *96.0%*	0.803	0.728	0.890
Verbal memory task	4.02 (2.14) *89.1%*	4.96 (2.36) *96.8%*	0.774	0.704	0.892
Inhibitory control	23.94 (8.46) *86.1%*	26.18 (8.08) *97.7%*	0.819	0.750	0.888

Overall Cronbach’s Alpha = 0.91; Variance explained by the first component was 55.8%.

^A^Subtest discarded after observing the principal component analysis results (factor loading <0.50).

**Table 4:** Principal components analysis and reliability analysis for Test B.

	Correct answers Mean (Standard Deviation) *% Correct response*		Reliability analysis	Principal component analysis	
	Patients	Controls		Corrected Item-Total Correlation	Cronbach’s Alpha if Item Deleted
Word categories task	8.15 (5.01) *79.8%*	11.64 (4.09) *96.0%*	0.726	0.635	0.885
Attention task I	2.97 (1.42) *82.1%*	4.67 (2.26) *84.8%*	0.251^A^	–	–
Auditory memory task	2.77 (1.81) *68.8%*	3.95 (2.38) *80.3%*	0.489^A^	–	–
Opposite task	23.77 (19.43) *64.6%*	26.18 (13.07) *92.3%*	0.639	0.528	0.892
Visual memory task II	13.23 (5.51) *81.8%*	18.05 (3.46) *98.6%*	0.686	0.611	0.887
Attention task II	15.72 (10.25) *91.1%*	21.03 (9.62) *98.8%*	0.817	0.779	0.875
Sequences	13.72 (8.63) *90.4%*	14.38 (3.57) *98.6%*	0.770	0.713	0.880
Calculus task	15.62 (9.26) *90.0%*	17.18 (6.53) *96.1%*	0.754	0.670	0.883
Visual memory task I	15.13 (3.83) *94.6%*	16.74 (3.41) *99.7%*	0.512	0.441	0.898^B^
Puzzles	1.08 (0.90) *100%*	2.49 (1.59) *100%*	0.712	0.628	0.885
Written Comprehension	13.77 (5.74) *88.7%*	15.69 (5.15) *98.9%*	0.742	0.677	0.882
Shopping Task	6.28 (4.14) *88.8%*	10.51 (4.28) *98.1%*	0.781	0.713	0.880

Overall Cronbach’s Alpha = 0.90; Variance explained by the principal component was 45.5%.

^A^Subtest discarded after principal component analysis (factor loading <0.50).

^B^Subtest discarded after internal consistency analysis (item-total correlation <0.50).

**Table 5:** Standardized total test scores after item selection.

	Test A	Test B
Cronbach’s Alpha	0.91	0.90
T-scores*		
Mean [95% Confidence Interval]		
Controls	50.00 [31.49; 68.05]	50.00 [27.50; 72.52]
MCI/Mild Dementia	9.03 [−7.20; −25.26]	−6.56 [−34.69; 21.84]
**Correlation with MMSE** score	0.39	0.52
Spearman’s R *(linear regression p-value)*	*(p* < *0.001)*	*(p* < *0.001)*
**Correlation with MoCA** score	0.52	0.62
Spearman’s R *(linear regression p-value)*	*(p* < *0.001)*	*(p* < *0.001)*
**Minimal important difference (MID)**	40.97	56.82
**Smallest real difference (SRD)**	37.89	43.04
**Difference of MID and SRD (%)**	7.5%	23.9%
**Area under the ROC curve**	0.74	0.75

MCI – mild cognitive impairment; ROC – Receiving operator characteristic; MoCA – Montreal Cognitive Assessment; MMSE – Cognitive Assessment and Mini Mental State Examination.

^*^Sum of the standardized subtest scores transformed to a T-distribution.

**Table 6:** Results from the test-retest study (consistency two way mixed single intraclass correlation coefficient).

	Subtest scores Mean (standard deviation)			ICC	
	Trial 1	Trial 2	Trial 3	2 trials	3 trials
Word categories task	14.81 (5.69)	15.06 (5.11)	15.80 (4.96)	0.797	0.836
Opposite task	30.80 (22.94)	32.06 (21.87)	34.18 (20.30)*	0.754	0.814
Visual memory task II	13.40 (3.01)	13.27 (2.91)	14.31 (3.29)	0.700	0.790
Attention task II	21.97 (8.78)	23.68 (8.50)	24.54 (7.40)	0.406	0.547
Sequences	11.28 (5.61)	10.96 (5.53)	11.31 (5.72)	0.795	0.855
Calculus task	19.64 (9.67)	20.35 (9.08)	20.52 (9.54)	0.847	0.880
Puzzles	4.97 (3.82)	5.04 (2.23)	5.02 (2.42)	0.610	0.768
Written comprehension	14.01 (5.18)	14.05 (3.78)	14.92 (3.61)*	0.660	0.811

^*^Statistically significant difference between trials 2 and 3 in Student’s T test for related samples (p < 0.05).
