# Supplementary material for: Development of a self-administered web-based test for longitudinal cognitive assessment
Source: Sci Rep. 2016 Jan 8;6:19114. doi: 10.1038/srep19114 (PMC4705487; doi:10.1038/srep19114)
Supplement: Supplementary Information [file srep19114-s1.pdf]

Brain on Track: development of a self-administered web-based test for longitudinal cognitive assessment

Luis Ruano MD, MPH[1,2,3]*, Andreia Sousa BSc[1], Milton Severo PhD[2,3], Ivânia Alves MD[1], Márcio Colunas BSc[4], Rui Barreto MD[1], Cátia Mateus BSc[1], Sandra Moreira MD[1], Eduardo Conde BSc[4], Virgílio Bento PhD[5], Nuno Lunet PhD[2,3], Joana Pais PhD[1.3], Vítor Tedim Cruz PhD[1,3.4]

1 - Department of Neurology, São Sebastião Hospital, Entre Douro e Vouga Hospital Center; Address: Rua Dr. Cândido de Pinho, 4520-211 Santa Maria da Feira, Portugal; Phone + 351 256379700

2 - Department of Clinical Epidemiology, Predictive Medicine and Public Health, University of Porto Medical School; Address: Alameda Prof. Hernâni Monteiro 4200-319 Porto, Portugal; Phone: +351 225513652

3 - EPIUnit - Institute of Public Health, University of Porto; Address: Rua das Taipas 135-139, 4050-600, Porto, Portugal; Phone: +351 222061820

4 - Clinical Research Office, Health Sciences Department, University of Aveiro; Address: Campus Universitário de Santiago, 3810-193, Aveiro, Portugal; Phone: +351 234370200

5 - University Institute of Maia – ISMAI; Address: Avenida Carlos de Oliveira Campos, 4475-690, Maia, Portugal; Phone: +351 229866000

Appendix 1- Subtest Description

Name	Target Domains	Subtest Description
Attention task I	Attention	A random number is shown on screen, then, random numbers appear on screen for 3 seconds and the participant should press the space key whenever the initial number is shown.
Auditory memory task	Declarative Memory<br>Language	A short story is read. Then, 3 questions about details of the story are asked and the participant must select the correct answer from 4 possible choices.
Puzzles	Constructive ability	The screen is divided into two parts: on the right the target image is shown, on the left several of the image composing pieces are scattered. The purpose of the task is to complete the image using the scattered pieces.
Visual memory task I	Visual memory	Several worms appear on screen in random sequence. The participant must memorize the order in which the worms appear and then click on the respective images to reproduce the sequence.
Shopping Task	Calculus	A shopping item is shown on screen with a price. The participant must click on a set of coins and euro notes to make the exact amount shown.
Attention task II	Attention<br>Language	A letter is shown on screen and then several screens are shown with multiple letters placed randomly. The patient is asked to identify and click on the initial letter.
Opposite	Inhibitory control<br>Executive functioning	In a central position of the screen, a large arrow is shown. The participant must press the keyboard arrow in the opposite direction to that shown by the large arrow.
Visual memory task II	Attention<br>Short term memory	On the screen, 3 cubes of different colors light up in a random sequence. The participant must memorize this sequence and reproduce it using the mouse to click on the cubes in the correct order.
Calculus	Calculus	The participant should perform the numerical calculation shown on screen and input the number via keyboard or by using the mouse to click on a keypad with numbers on screen. The operation should be completed before a balloon reaches the top of the screen.
Sequences	Executive function<br>Abstract thought	The upper part of the screen displays a set of figures that follows a certain logic sequence. The participant should select the figure that completes the sequence from 4 possible figures.
Written Comprehension	Language<br>comprehension	On the screen, there are several sets of geometric objects of different shapes and colors. The participant must select the set that matches the description of the written command.
Word categories	Language	The participant must select the correct category for the word that is shown on the screen by dragging the word to the corresponding box. If the word does not belong to any of the categories, the participant must drag it to the garbage can.
Inhibitory Control	Language<br>Inhibitory control	A colored circle and the name of a color are shown on screen, and two buttons: YES/NO. The color of the circle and the color of the word font are random. In the first set, the participant must select YES when the word and the color of the circle are the same, and NO when they are different. In the middle of the subtest, a new instruction appears on screen, and now the participant must select YES when the color of the circle matches the color of the word font.
Verbal memory	Short term memory	The participant is asked to memorize a list of 4 words. After a short delay, 10 words are shown on screen (4 correct and 6 distracters) and the participant must click on the correct words.

Appendix 2 - Detailed demographics of patients from Study I

			Education group				
			1-4	5-9	10-12	>12	Total
**Test A**	**Age group**	40-59	1 (2.0%)	2 (4.1%)	0 (0.0%)	0 (0.0%)	3 (6.1%)
		50-59	2 (4.1%)	3 (6.1%)	1 (2.0%)	0 (0.0%)	6 (12.2%)
		60-69	6 (12.2%)	1 (2.0%)	0 (0.0%)	0 (0.0%)	7 (14.3%)
		70-79	21 (42.9%)	3 (6.1%)	0 (0.0%)	1 (2.0%)	27 (55.1%)
		80-89	6 (12.2%)	0 (0.0%)	0 (0.0%)	0 (0.0%)	6 (12.2%)
		Total	38 (77.6%)	9 (18.4%)	1 (2.0%)	1 (2.0%)	49 (100.0%)
**Test B**	**Age group**	50-59	1 (2.6%)	0 (0.0%)	0 (0.0%)	0 (0.0%)	1 (2.6%)
		60-69	9 (23.1%)	0 (0.0%)	1 (2.6%)	1 (2.6%)	11 (28.2%)
		70-79	17 (43.6%)	3 (7.7%)	0 (0.0%)	0 (0.0%)	20 (51.3%)
		80-89	7 (17.9%)	0 (0.0%)	0 (0.0%)	0 (0.0%)	7 (17.9%)
		Total	34 (87.2%)	3 (7.7%)	1 (2.6%)	1 (2.6%)	39 (100.0%)
